# WHAT DOES INTEGRATION LOOK LIKE IN MIXED-METHODS RESEARCH?

**DOI:** 10.1093/geroni/igae098.1490

**Published:** 2024-12-31

**Authors:** Pamela Saunders

**Affiliations:** Georgetown University, Washington, District of Columbia, United States

## Abstract

Multi-methods and mixed-methods research usually include both qualitative and quantitative data to gain a more holistic understanding of a research question. Multi-method research involves a variety of data sources and methods. A defining feature of Methods is the potential integration at multiple levels, including schools of thought, data, data collection methods, or analysis. The Friendship Study (de Medeiros et al., 2012) used a mixed-methods approach to examine perceptions of friendship among people living with dementia. The participants included ten staff members and 31 assisted living residents with moderate to advanced dementia. The first step was to define ‘social interaction’ and how it relates to the larger construct of friendship. Staff completed a matrix indicating their perceptions of which residents were friends. We conducted 6-months of direct observation with detailed field notes. The integration process focused on data collection methods, including interviews and surveys with staff and field notes describing direct observations of interactions between residents. Findings showed that the qualitative data from staff did not support the qualitative data from field notes of direct observation. This paper dives deeply into integrating these qualitative and quantitative data and analyses. A summary of specific integration techniques will be outlined.

